# How and why are communities of practice established in the healthcare sector? A systematic review of the literature

**DOI:** 10.1186/1472-6963-11-273

**Published:** 2011-10-14

**Authors:** Geetha Ranmuthugala, Jennifer J Plumb, Frances C Cunningham, Andrew Georgiou, Johanna I Westbrook, Jeffrey Braithwaite

**Affiliations:** 1 Australian Institute of Health Innovation, University of New South Wales, Sydney, NSW 2052, Australia

## Abstract

**Background:**

Communities of Practice (CoPs) are promoted in the healthcare sector as a means of generating and sharing knowledge and improving organisational performance. However CoPs vary considerably in the way they are structured and operate in the sector. If CoPs are to be cultivated to benefit healthcare organisations, there is a need to examine and understand their application to date. To this end, a systematic review of the literature on CoPs was conducted, to examine how and why CoPs have been established and whether they have been shown to improve healthcare practice.

**Methods:**

Peer-reviewed empirical research papers on CoPs in the healthcare sector were identified by searching electronic health-databases. Information on the purpose of establishing CoPs, their composition, methods by which members communicate and share information or knowledge, and research methods used to examine effectiveness was extracted and reviewed. Also examined was evidence of whether or not CoPs led to a change in healthcare practice.

**Results:**

Thirty-one primary research papers and two systematic reviews were identified and reviewed in detail. There was a trend from descriptive to evaluative research. The focus of CoPs in earlier publications was on learning and exchanging information and knowledge, whereas in more recently published research, CoPs were used more as a tool to improve clinical practice and to facilitate the implementation of evidence-based practice. Means by which members communicated with each other varied, but in none of the primary research studies was the method of communication examined in terms of the CoP achieving its objectives. Researchers are increasing their efforts to assess the effectiveness of CoPs in healthcare, however the interventions have been complex and multifaceted, making it difficult to directly attribute the change to the CoP.

**Conclusions:**

In keeping with Wenger and colleagues' description, CoPs in the healthcare sector vary in form and purpose. While researchers are increasing their efforts to examine the impact of CoPs in healthcare, cultivating CoPs to improve healthcare performance requires a greater understanding of how to establish and support CoPs to maximise their potential to improve healthcare.

## Background

Improving productivity was one of three strategies put forward as a means of addressing the funding shortfall projected for the National Health Service (NHS) in the UK for 2011-2017 [1]. Funding shortfalls are not exclusive to the NHS; health services across the world are faced with the need to deliver high-quality care within economically constrained environments. Improving productivity in the healthcare sector means adding value to how resources are used to deliver high-quality healthcare that meets the needs of the people - that is, to deliver high-quality healthcare effectively. To address this need, the sector has looked to other industries for strategies to improve organisational performance. One such strategy has been the promotion and fostering of communities of practice (CoP) that have gained recognition in the business sector for improving organisational performance [2,3].

In business, CoPs are promoted as drivers of knowledge management, as a mechanism for the sharing of tacit knowledge, sparking innovation, reducing the learning curve for new staff, and as a means of creating social capital and adding organisational value [2,4]. These claims have led to CoPs being promoted in healthcare as a tool to enhance knowledge and improve practice [5]. Ostensibly, they provide a means for knowledge to cross boundaries, generate and manage a body of knowledge for members to draw on, promote standardisation of practice, and "innovate and create breakthrough ideas, knowledge, and practices" [4]. However, little is known about the organisational processes that lead to the successful creation of knowledge-based structures such as CoPs [6].

The term CoP was originally proposed by Lave and Wenger in 1991 as a central element in their theory of 'situated learning', based on the observation that learning was more than acquiring knowledge; it involved a complex relationship between a novice and expert, peripheral participation in practice, being socialised into the practice and developing an identity within the practice community [7,8]. In 1998, the concept was refined by Wenger to extend beyond the novice-expert relationship by focussing more on the interaction between individuals and the participation of people who are engaged in creating and sharing knowledge [9,10] Three dimensions were proposed as defining a CoP: joint enterprise (what it is about); mutual engagement (the interactions that lead to the shared meaning); and a shared repertoire (of resources such as techniques, tools, experiences or process and practice). In 2002, Wenger and colleagues redefined CoPs in terms of a managerial tool which would bring together groups of people working in parallel to share knowledge and to innovate. Characterised by a shared *domain *of interest, a *community *that pursues the shared interest, and *practice *or shared repertoire of resources, CoPs were defined as:

*"... groups of people who share a concern, a set of problems or a passion about a topic and who deepen their knowledge and expertise in this area by interacting on an ongoing basis... These people don't necessarily work together on a day-to-day basis, but they get together because they find value in their interactions, as they spend time together, they typically share information, insight, and advice. They solve problems. They think about common issues. They explore ideas and act as sounding boards to each other. They may create tools, standards, generic designs, manuals, and other documents; they may just keep what they know as a tacit understanding they share... Over time, they develop a unique perspective on their topic as well as a body of common knowledge, practices and approaches. They also develop personal relationships and established ways of interacting. They may even develop a common sense of identity. They become a community of practice." *[7:p4-5]

In redefining CoPs in 2002, Wenger and colleagues argued that organisations need to actively and systematically cultivate CoPs for their benefits [7]. If CoPs are to be cultivated, there is a need to examine how they have been applied and to assess their impact in improving healthcare practice. Li and colleagues systematically reviewed the literature published between 1991 and 2005 to examine the evidence on the effectiveness of CoPs in the healthcare sector [11]. Having found no studies that met their inclusion criteria for quantitative studies, they concluded that the effectiveness of CoPs in the healthcare sector remained unclear. Li and colleagues reported that CoPs varied considerably in the way they functioned and were structured [11], which is in keeping with the way that Wenger and colleagues redefined CoPs in 2002 [7]. Given these variations in form and structure, and the lack of evidence demonstrating their effectiveness in healthcare, there is a need to further examine and explore the application of CoPs in the sector and to study the relationship between how CoPs are established and their impact in improving performance of healthcare organisations.

To this end, a systematic review of the peer-reviewed health and medical literature was undertaken to explore how and why CoPs have been established in healthcare. The following questions were examined:

• What was the purpose of establishing CoPs in healthcare?

• What was the composition of these CoPs?

• How have members of health sector CoPs interacted and communicated with each other and exchanged information or knowledge? and

• Have CoPs demonstrably improved performance of healthcare organisations?

This review is an important step in improving our understanding of CoPs as they currently operate in healthcare. It is a starting point to understanding the role of CoPs in improving performance of healthcare organisations; and will ultimately inform the design of a framework to systematically evaluate CoPs for their effectiveness in improving practice and their capability to sustain improved practice initiatives [12,13].

## Methods

In October 2009 the electronic databases MEDLINE, CINAHL, EMBASE, Web of Science and EconLit were systematically searched for primary research studies and systematic reviews on CoPs published between 1 January 1990 and 30 September 2009. To capture the diversity of the evolving concept of CoPs, while maintaining a focus on the exchange and acquisition of knowledge and the learning that is common ground to all definitions of CoPs, the following search terms were used: *community/communities of practice, community/communities of interest, community/communities of learning, community/communities of knowledge, learning community/communities, knowledge community/communities or situated learning*. These search terms were developed by members of the research team, using an iterative process that included review of seminal and emerging literature and discussion with a convenience sample of CoP sponsors and founders. The search was limited to research on human subjects and papers published in the English language.

For the purpose of this study, we defined improved performance of healthcare organisations to mean a demonstrated (as opposed to a self-reported) change in behaviour or work practice, or an improvement in process or clinical outcomes as demonstrated by a change in a performance indicator, which could be attributed to participating in a CoP activity or to accessing resources provided by the CoP. This was a definition developed by the research team for the overarching project on evaluating CoPs in healthcare [13]. For this reason, this review was limited to empirical research and case-studies with a focus on CoPs or situated learning involving practitioners in the healthcare sector. Only papers published in peer-reviewed journals were included. Exclusion criteria were as follows:

- Studies reporting on CoPs in sectors other than healthcare.

- Studies reporting on CoPs whose members were not directly involved in delivering healthcare, such as those focussed on activities presented as medical education; community based learning; classroom and undergraduate teaching, learning and curriculum development; student residential learning communities; or the pharmaceutical industry.

- Records with no abstracts, unless it was clear from the title that the paper was relevant.

- News-style or opinion articles, theses and dissertations, and abstracts of conference proceedings without full peer-reviewed papers.

Two authors (GR and JJP) independently reviewed all identified abstracts using the selection criteria, eliminated duplicates, and shortlisted abstracts for retrieval of paper and detailed review. When decisions differed, a final decision was made after discussion between the two reviewers. One author (GR) reviewed and extracted using a standardised template, required information from all retrieved papers except for those that employed ethnographic methods; these were reviewed by JJP. When relevance of the paper was uncertain, or the findings were difficult to extract, both authors independently reviewed the paper and reached a conclusion.

## Results

**Figure 1:**
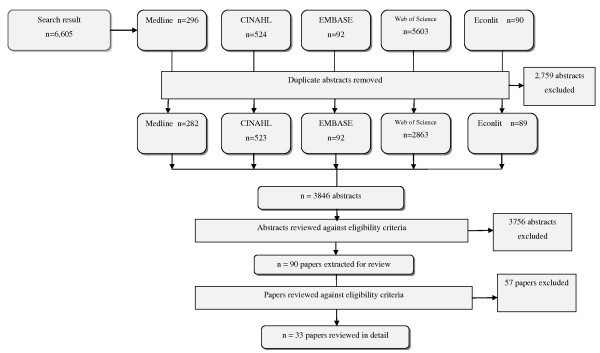
**Process of extracting, identifying and reviewing literature on communities of practice in the healthcare sector**.

**Figure 2:**
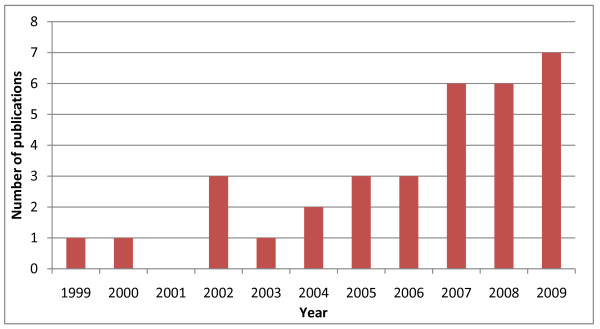
**Peer-reviewed publications on communities of practice in the healthcare sector, by year of publication**.

The search strategy identified 6,605 abstracts, of which 90 were potentially eligible for inclusion, based on the selection criteria. Review of the manuscripts eliminated a further 57 papers that did not meet inclusion criteria, leaving 33 papers for detailed review (see Figure 1). The earliest empirical research on a CoP in the healthcare sector identified by the search strategy, and included in the detailed review, was published in 1999; but more than half (19 of the 33 papers reviewed) were published in or after the year 2007 (Figure 2). Two systematic reviews on CoPs pertaining to the healthcare sector were published in 2009, examining the effectiveness of collaboration models in cancer surgery [14] and the use of CoPs in the health and business sectors [11].

### Country of origin

The United Kingdom (UK) was the most frequent country of origin, with 13 papers reporting on research undertaken in the UK [15-27]. Seven of the papers reported on research from Canada [28-34]; five from the United States of America (USA) [35-39]; four from Australia [40-43]; one from Denmark [44]; and one that included participants from the USA, Canada and Australasia [45]. The two systematic reviews on CoPs were conducted by researchers in Canada [11,14].

### Composition and intended purpose of establishing CoPs in healthcare

In order to explore whether the composition of the CoP reflected its intended purpose, these two aspects were examined simultaneously. Composition was examined in terms of whether or not the CoP members were from the same organisation or profession. Intended purpose was examined in relation to the focus on i) learning and exchange of information and knowledge, or ii) sharing and promoting evidence-based practice. The findings are summarised in Table 1 in chronological order, to study changing trends in the purpose of establishing CoPs, their composition, and to reflect the evolving concept of CoPs. The majority (25 of the 31) of the primary research papers extracted for this review reported on CoPs that involved more than one profession or organisation. Within this composition, the number of papers reporting on CoPs that focused on the two categories of intended purpose was almost equal, while the single profession or organisation CoPs were more focussed on changing practice or implementing evidence-based practice. Irrespective of whether the CoPs involved single or multiple professions or organisations, in keeping with the evolving CoP concept, the earlier publications focussed on learning and exchange of information and knowledge, while the later publications focussed on changing or improving practice.

**Table 1:** Composition and purpose of establishing communities of practice in the healthcare sector, by setting and in chronological order of publication*

Year paper published	Settings	**Why was the CoP established or what relevance did the CoP have to the research?**^**†**^	Reference
**A. Multi-organisation or multi-professional (n = 25)**			

*a. Learning, information and knowledge exchange (n = 12)*			

1999	Ten hospital and community-based healthcare organisations	The CoP concept was used to explore the process by which novice clinicians acquired competencies.	[27]

2002	Urologists, radiation oncologists, physicians and nurses delivering in-hospital cancer treatment.	CoPs were used as a tool to enrol key professionals and create, mobilise, diffuse and integrate knowledge relating to a radical innovation.	[24]

2002	Agencies involved in the delivery of local services for the elderly, and providers of dental and ENT services.	CoPs were established to help facilitate inter-agency collaboration.	[20]

2003	Members from agencies involved in delivering local services for the elderly, and service users.	The construction and work of two multi-stakeholder CoPs was facilitated to understand the acquisition and use of knowledge to help improve services.	[16]

2004	General practitioners, practice nurses and associated medical staff in two general practices	Researchers set out to understand how clinicians derive and use knowledge in practice. The fact that clinicians relied on their CoPs to obtain information was a finding.	[17]

2005	Anaesthetic teams consisting of novice or trainee nurses and doctors, and experienced operation-department practitioners and consultants.	The concept of legitimate peripheral participation in CoPs was used to explore the distribution of work and knowledge within anaesthetic teams.	[18]

2006	Researchers, practitioners, and policy makers with interests in web-assisted tobacco interventions.	A group of diverse professionals from geographically-dispersed locations were brought together to lay the foundations for a CoP.	[45]

2006	Anaesthetists from ten anaesthetic departments.	The online system created a CoP within which participants could anonymously post critical incidents for discussion.	[22]

2007	Collaborative relationship between the Society of Obstetricians and Gynaecologists, hospital insurance provider, and/or the provincial government and participating hospital.	The obstetric patient-safety program was based on principles of team effort, CoPs and organisational behaviour.	[32]

2008	Clinical nurse consultants, educators and managers of intensive care units.	The email Listserv led to a sense of community and the creation of a CoP, facilitating the exchange of information.	[42]

2008	Senior clinical managers	Emergence of CoPs was one of many effects on clinical practice reported in the paper.	[23]

(Note: Reference number 23 has been included in the multi-organisation or multi-professional category for the following reasons: i) The authors stated that the general composition of the clinical leadership program was 10% allied health professionals and 90% senior clinical nursing staff; ii) The authors also stated that the leadership program had, over three years, supported over 100 staff working for NHS Lanarkshire. Given that there is more than one hospital within this county, it is possible that the program participants were not necessarily co-located within the one service unit.)			

2009	Clinicians working in nine rural and two urban paediatric emergency departments.	A virtual community of practice was established to facilitate knowledge exchange.	[29]

*b. Sharing and promoting good practice/evidence-based practice (n = 13)*			

2004	Healthcare workers and researchers with an interest in evidence-based care.	Virtual CoPs emerged spontaneously as people identified common interests.	[21]

2004	State and local public health agencies engaged in child health-information system-integration projects.	A CoP was created to bring together a diverse group of professionals from geographically-dispersed agencies to learn from each other, to capture best practices and to collaboratively address challenges.	[39]

2005	Practising nurses in gerontology and academics (Nursing Demonstration Project).	The CoP provided a tool to bridge the divide between practising nurses and academics. The CoP was also involved in developing best-practice statement methodology and in designing a virtual college.	[19]

2005	Practising nurses in gerontology and academics (Nursing Demonstration Project).	The potential of the CoP and the virtual college to accelerate the achievements of evidence-based practice was explored.	[46]

2006	Nine healthcare systems and ten hospitals represented.	The intervention to reduce hospital-acquired infections was multifaceted and included developing a CoP.	[37]

2007	Emergency department clinicians from multiple hospitals. CoP is partnership between the ED clinicians and the National Institute of Clinical Studies, which provides implementation expertise and support.	An emergency department (ED) collaborative was established and was successful in engaging clinicians from 47 hospital ED teams from across the country. This led to a network of clinicians interested in improving uptake of evidence, leading to the establishment of an ED CoP. The CoP acted as a mechanism that built on the knowledge and expertise of the clinicians to implement evidence-based practice.	[40]

2007	Representatives from the family physician, physiotherapy and occupational therapy licensing Boards; and clinician associations, observers from the compensation board and its research institute. Experts and opinion leaders on low back pain. Scientific committee.	A CoP approach was used to develop clinical guidelines.	[34]

2007	Researchers and program providers who work on improving telephone-based counselling for smoking cessation.	The CoP model was used to improve telephone-based counselling for smoking cessation.	[31]

2008	Journal club and case conferences attended by physicians and other clinicians from Internal Medicine, Neuroradiology, Anaesthesiology, Otology/Head and Neck Surgery, Dermatology, And Ophthalmology Departments.	The CoP concept was used to structure continuous medical education accredited journal clubs and case conferences to be interactive and problem-based, with the objective of increasing the likelihood of physicians implementing evidence-based care.	[36]

2008	Diverse stakeholders, including hospitals, non-profit organisations and city agencies, working together to improve cancer screening in community health centres.	The community health centres did not have the capacity to provide care for people with abnormal screening tests and cancer diagnosis, nor did they have partnerships with available community resources. Local CoPs were established to address this gap. Regional CoPs were established to provide forums on a wider scale geographically, for sharing ideas, identifying resources, and encouraging action on local community building efforts.	[38]

2008	Five acute hospital wards, six home-care, and seven day hospitals.	The intervention to promote evidence-based practice included membership of a CoP.	[26]

2009	Health and social-care communities to address problems with discharge planning and transfer of care.	CoP was established to test whether the bringing together of a wide range of staff, with a shared interest, would make a meaningful contribution to sustainable service improvement.	[15]

2009	Children's mental health practitioners (frontline social workers, child and youth workers) working in six service-provider organisations, newly-mandated to use the standardised outcome measurement tool.	Support structure provided to help implement the adoption of an electronic version of a standardised outcome measurement tool included access to a CoP.	[28]

**B. Single-organisation or single profession (n = 6)**			

*a. Learning, information and knowledge exchange (n = 2)*			

2000	Nurses with little research experience	The workshop provided the nurses access to a CoP where they could work with experienced researchers.	[35]

2002	Small group of physicians	The concept of CoP was used to examine the learning that occurred within small groups of physicians.	[33]

*b. Sharing and promoting evidence-based practice/promoting innovation in clinical practice/supporting clinical practitioners (n = 4)*			

2007	Occupational therapists working in a large metropolitan hospital.	The CoP was proposed as a tool to support occupational therapists reflecting on how their profession is conceptualised and described, and to define their unique contribution to patient care within a biomedically-dominated institutional context.	[43]

2007	Hospital setting.	The clinical planning group had characteristics of CoPs.	[44]

2008	Cancer surgery.	The CoP was established and endorsed as a means of facilitating quality improvement.	[30]

2009	General practitioners.	CoP was established to address the quality of referral letters.	[41]

**C. Systematic reviews (n = 2)**			

2009	Healthcare sector.	Systematic review of CoPs in business and healthcare sectors.	[11]

2009	Regional collaborations and CoPs within the surgical settings.	The rationale for undertaking the systematic review was the need to investigate whether the CoP concept could be implemented through collaborative initiatives.	[14]

* Studies have been presented in chronological order to study the trend in the composition and emphasis of the CoP reflecting the evolving concept of CoP.

^**† **^The research did not always involve establishing a CoP. Some researchers applied the CoP concept to examine and understand existing groups.

CoP = Community of Practice

### Means of interaction, communication and exchanging information or knowledge

Lack of consistency in reporting made it difficult to identify and compare the various methods used by members of CoPs to communicate, interact and share information and knowledge with each other. Based on information provided in the papers, 16 of the 31 primary research studies reported that CoP members met face-to-face on at least one occasion (Table 2). While these papers made particular reference to face-to-face means of interaction, it was not possible to know whether other means of communication (such as email) were used as well. Email and web-based systems were the next most popular method.

**Table 2:** Methods available for members of communities of practice to interact and communicate with each other and to exchange information or knowledge, in chronological order of publication*

			Activities and methods of communication/interaction			
Reference and year of publication	Workshops	Seminars	Meeting of members	Emails	Web-based systems and blogs	Other
[27] 1999						Face-to-face ongoing interactions at place of work.

[35] 2000	A one-off workshop based on situated-learning model was organised to engage nurses in research.					

[20] 2002			Regularly, to move the project goals forward			

[33] 2002			At least 6 - 10 times a year.			

[24] 2002		Coordinated information, training and education sessions for medical professionals and other stakeholders.				

[16] 2003			Each CoP met seven times during the study period.			Members undertook 'homework' to seek information in between meetings.

[17] 2004			Meetings of practice staff, GPs, partners, executives, admin staff, partners and practice manager, and practice award nurse team.			Multiple informal gatherings and discussions, patient-doctor consultations, home visits.

[21] 2004				Targeted email and networking service for health practitioners and researchers.		

[39] 2004			Face-to-face meetings.	Listserv	Interactive website.	Site-visits, each lasting 2.5 days; followed by circulation of a newsletter summarising the meeting and a CD compilation of the presentations made during the site visits. Teleconferences. Opportunity to participate in special projects funded by the sponsoring agency.

[18] 2005						Face-to-face ongoing interactions at place of work.

[19] 2005			Met for 2 days at the start of the project, and at months 4, 9 and 14.		Participants were encouraged to participate in virtual workshops to discuss models or descriptions of gerontological nursing identified from the literature.	

[46] 2006					Virtual college.	

[45] 2006			The initial bringing together of the group.	Email discussions		

[37] 2006			Met monthly the project to report and share and effective strategies, contribute to problem reinforce and and to ensure consistency in data collection.			Presentations of evidence-base by experts at the kick-off session. Monthly progress reports posted on bulletin boards.

[22] 2006					Online system for posting and discussing critical incidents in anaesthesia.	

[40] 2007	Not described in detail. The authors make reference to the program functioning predominantly through a virtual platform with opportunity for personal communication and networking.					

[31] 2007		Regular web-based seminars	Occasional face-to-face meetings			Teleconferences. CoP members were granted access to online resources including policy, program and research aids; including standard research and evaluation protocols.

[34] 2007			Symposium to discuss the recommendations.		Guidelines were presented to members of the CoP by postal mail, email, and website. Web-based system was offered as a method of communication.	

[44] 2007			While not explicitly stated in the paper, it is assumed that the activities undertaken by the group would have involved face-to-face interaction.			The clinical planning group was established to plan clinical training, coordinate installation of training versions of EMP, and organise a range of practical tasks.

[32] 2007	Multidisciplinary workshops				Web-based platform used to deliver educational content.	

[43] 2007			Exchanges during routine work.			

[42] 2008				Email Listserv		

[36] 2008			Attendance and participation in journal clubs and case conferences.			

[26] 2008					Knowledge-pooling and translation is facilitated through a virtual practice-development college.	

[30] 2008	Not described in the paper.					

[38] 2008			Local and regional meetings.			Teleconferences with community health centre teams.

[23] 2008			Leadership program that led to the emergence of CoP included group reflection exercises.			

[15] 2009	Bi-monthly, half-day workshops to discuss 'hot topics' identified by the 'core' group.				Database of members published on a CoP website.	

[29] 2009					12 case-based learning modules with content relevant to clinical topic, and an asynchronous online discussion board.	

[41] 2009				Project coordinator maintained regular communication with CoP members by email and telephone.		

[28] 2009	3-day training program		Face to face meetings, site visits for individualized consultation.	Email support	Web and wiki support.	Reliability and software training, telephone support, information provided on the website, quarterly agency reports.

[11] 2009	Not applicable - systematic review of the literature					

[14] 2009	Not applicable - systematic review of the literature					

*Studies have been presented in chronological order to study the trend in activities and methods of communication as the concept of CoPs evolved over time.

CoP = Community of practice

Communication and exchanges for some groups occurred predominantly in their usual work environment. This was particularly so in the case of existing groups examined using the CoP concept [17,18,27]. Others were established or developed as virtual communities [21,22,26,29,42]. However, most CoPs used a combination of methods for members to communicate and interact with each other. None of the primary research studies examined the methods of communication and interaction in relation to the impact of the CoP in achieving its objective. One case study did, however, identify the fact that the CoP was highly dependent on face-to-face meetings to keep the energy alive [39]; while in another CoP, the lack of opportunity for members to meet face-to-face in a quantitative intervention trial was explored as a possible explanation for why only a third of initially-recruited participants completed the necessary audit cycle [41].

### Improving healthcare performance

To examine the outcomes achieved by establishing or facilitating CoPs in healthcare, the research methods used to study CoPs and the findings or conclusions from the 33 research papers reviewed are summarised in Additional File 1. Of the 31 primary research papers included in the review, 24 utilised qualitative methods: collecting data through ethnographic observations and interviews; and content analysis of emails, discussion forums and reports. The majority of these studies used a single approach (such as interviews or observations) to gather data from a single source, providing little scope for validating the findings or obtaining a more comprehensive assessment of the value of the CoP. A more rigorous triangulation approach was adopted by Gabbay and colleagues, where data were collected from multiple sources (through non-participant observation, tape-recording of CoP meetings and subsequent analytic reflections, interviewing CoP members and reviewing CoP notes and output) as a means of obtaining a more comprehensive understanding of the value of CoPs in improving services for the elderly [16]. Other studies that utilised multi-methods to collect data included: Gabbay and le May [17] collecting data through non-participant observation, semi-structured interviews and document review to explore decision-making by primary care physicians; Russell and colleagues using interviews and tracking of email messages to explore knowledge-exchange processes [21]; Sharma combining observations with semi-structured interviews to collect information required to establish an online incident reporting system [22]; and Bossen using a combination of observations and interviews to gain insight into the process of implementing information technology in healthcare [44].

Three of the qualitative primary research studies explored the impact of CoPs in improving or changing practice. The first of these published in 2007 described the history and establishment of a CoP to help implement evidence-based practice in emergency departments [40]. Included in this case-study was a summary of the first major implementation activity initiated through the CoP, reporting statistically significant improvements (determined through audits) in target process indicators in mental health (see Additional File 1). In the second study published in 2008, clinicians attending continuing medical-education accredited journal clubs and case conferences (structured on the CoP concept) self-reported that 55% of the 200 'learnings' acquired through attendance at journal clubs and case conferences had been implemented [36]. Implementation was however not verified by the researchers reporting the study. A third qualitative research study published in 2009 explored whether establishing CoPs would help achieve sustainable service improvements [15]. The authors identified that the benefits of establishing CoPs could not be directly translated to service improvements.

The search strategy identified seven primary research studies that used quantitative research methods, in an attempt to determine the value of CoPs in the healthcare sector. While these seven studies are included in Additional File 1, the study design, outcome and findings of these quantitative studies are presented in more detail in Table 3. The seven studies included six intervention trials and one case study.

**Table 3:** Summary of the quantitative primary research studies included in the review

Reference and year of publication	Study design	Outcome measure	Findings
[37] 2006	Component of a randomized controlled trial. Intervention = multifaceted. Randomisation determined whether the intervention was to begin in the operating room or in the intensive care unit (and not to assign the patient to a study group). Project leaders and teams were established to implement evidence-based practice to reduce central line infections.	Adherence to evidence-based process indicators, as a proportion of CR-BSI reported during the previous year. Catheter-related blood-stream infections (CR BSI).	Process adherence increased from 0% to 85%. CR BSI dropped by more than 50% (from 1.7 to 0.4 per 1000 line days, p < 0.05). The success of this intervention across nine healthcare systems and ten hospitals was attributed by the authors to the direct involvement of the hospital leadership (within each hospital) in marketing and promoting the intervention and the development of local CoPs.

[45] 2006	Case-study of the establishment of a CoP following the bringing together of individuals known to work in the area of web-assisted tobacco intervention.	Potential emergence of a CoP.	Social network methods were used to demonstrate the establishment of networks following the initial meeting.

[32] 2007	Intervention trial. Intervention = multifaceted Managing Obstetric Risk Efficiently (MORE) program. Implementation at each of the 28 hospitals was led by a core inter-professional team.	Core clinical knowledge assessment. Culture change assessed using a culture change assessment tool. Frequency of liability claims and liability carrier (hospital) incurred costs.	Clinical core knowledge increased significantly, demonstrated by increase in test scores following completion of training modules. Improvement in the six elements - empowering people, learning, open communication, patient safety, teamwork, valuing individuals - was demonstrated using a culture change assessment tool developed for the program. In all of the 28 hospitals that provided data, the frequency of liability claims dropped over a three-year period, and liability carrier (hospital) costs showed a decreasing trend compared to pre-MORE program. This is in contrast to all other healthcare services, which showed a trend towards increase in costs. The development and annual operating costs were recovered by the end of three years.

[38] 2008	Intervention trial. Intervention = Regional cancer-collaborative to implement a regional approach to learning. Care-process leaders worked with teams to plan and implement practice change. Regional CoPs were established as a forum for sharing ideas, identifying resources, and encouraging action. Establishment of regional and local CoPs was encouraged.	Process evaluation of implementation activities. Breast, cervical and colon cancer screening rates.	Some processes were more difficult to implement than others, and implementation was easier at some sites and not others. Three of the four participating organisations implemented local CoPs. Screening documentation increased with all four cancers. Colon cancer screening-rates increased from 8.6% to 21.2%. This increase was seen in 3 of the 4 sites (the 4^th ^showed a drop). Authors concluded that improvements may be achieved in carefully selected organisations.

[26] 2008	Intervention trial. Intervention = multifaceted. The Caledonian Model designed to promote evidence-based practice included membership of a CoP.	Impact on nursing practice was assessed by baseline and post-intervention audits of policies, resources and education. The revised nurse-working index was used to explore perceived impact of the model on the nurses' work.	Facilities' audit results demonstrated improved practice through development of local guidelines and policies; use of validated screening tools; implementing guidelines; and ongoing training for staff. Patients' audits demonstrated more relationship-centred approach to care-provision; improved recording of patient and family feelings and expectations; assessment of individual needs; risk-screening; and greater involvement of the patient in decision-making. The authors acknowledge the limitations imposed by their inability to control for confounding events occurring concurrently.

[41] 2009	Intervention trial. Intervention = CoP established to improve standards in general practice, focussing specifically on quality of referral letters written to specialists.	Quality of letters written by GPs, scored using benchmarks established by members of the CoP.	Only five of the 15 recruited GPs completed the study; 102 referral letters were submitted by these 5 GPs. Statistically significant improvements in scores were reported from the scoring of the history and examinations components in the referral letters.

[28] 2009	Randomised trial. Participants randomised to CoP-supported or practice-as-usual arm of trial.	Content knowledge on assessment tool; self-reported change in practice; use of the Child and Adolescent Functional Assessment Scale (CAFAS) tool; and use of, and satisfaction with, implementation support.	The difference between the CoP and practice-as-usual groups, in terms of self-reported practice change, was not statistically significant. However, the CoP group demonstrated greater knowledge of the assessment tool at the end of the 12 months and greater use of the tool compared to the practice-as-usual group. The authors conclude that CoPs may be a useful strategy for promoting the implementation of evidence-based practice; but caution against generalisation, due to small size of the sample and one-year follow-up period.

In three of the six intervention trials, the interventions were multifaceted and included the establishment of, or participation in a CoP [26,32,37]. These three studies reported improved outcomes including improvements in developing local guidelines and policies, improved assessment of nutritional needs of older persons, increased use of screening tools, and greater involvement of the patient in decision making [26]; reduced frequency of insurance liability claims received by hospitals [32]; and improved rates of adherence to evidence-based process indicators [37]. The multifaceted nature of these interventions made it difficult to differentiate the impact of the CoP component of the intervention from the rest.

The interventions in the three remaining studies were largely based on the CoP concept. The outcome of interest in one of these studies was cancer screening rates [38]. While screening documentation and rates increased, the authors concluded that improvements may be achieved in carefully selected organisations. The second of these three studies established a CoP to improve standards in general practice, focussing specifically on the quality of referral letters written to specialists [41]. While quality improvement was reported, caution is required in assessing the impact due to a high attrition rate. The third study was a randomised trial where the impact of CoPs in promoting uptake of evidence-based practice was explored [28]. This was the only randomised controlled trial that the search strategy yielded. While self-reported change in practice was not demonstrated, the CoP group demonstrated greater knowledge of the assessment tool and actual utilisation of the tool in practice at the end of the 12-month study period.

Two systematic reviews of the literature on CoPs in the healthcare sector were also published in 2009 [11,14]. One of the objectives of the systematic review published by Li et al was "to assess the evidence on the effectiveness of CoPs in healthcare settings." [11]. They identified, and reviewed in detail, 13 papers reporting primary studies in the health sector. Due to the different search strategies that included different databases and search terms, and also the significant increase in peer-reviewed publications on CoPs since the end of the review period for the Li paper in 2005 (see Figure 2), only five of the 31 independent studies identified by our search were included in the Li review. No papers were found to meet their eligibility requirements for quantitative analysis, leaving the authors to conclude that the effectiveness of CoPs in healthcare remained unclear. Given that the Li review period ended in 2005, and that much of the research on CoPs in healthcare has been published since 2005 (Figure 2), the individual studies in the Li review were not extracted and examined individually.

The second review by Fung-Kee-Fung and colleagues included papers published up to July 2006 [14]. This review was limited to research on the use of regional collaborations in surgical practice. The authors identified and reviewed seven papers. Effectiveness was assessed through process indicators such as compliance with evidence-based care processes; establishment of a database as a process to assist clinicians to be proactive in improving clinical care; compliance with program standards; and by measuring attitude change in clinicians. One study examined mortality rates; and two studies reported changes in clinical practice in line with regional guidelines, observed over a one-year period; and changes in medication prescription at a two-year follow up. Based on improvements in performance measured using these indicators, the authors concluded that sustainable collaborative programs can be realised through initiatives such as CoPs "that are regional in scope, evidence-based, data-driven, and supported institutionally through strategic partnerships that provide comprehensive support as part of the philosophy of continuous quality improvement." [14] As these studies had been reported and published as quality improvement collaboratives and not CoPs, they were not extracted by our search strategy. The original papers from the Fung-Kee-Fung review were not retrieved and reviewed due to the narrow focus on collaborative initiatives in the surgical setting.

## Discussion

A systematic search and review of the peer-reviewed literature on CoPs in the healthcare sector has identified that that there is much diversity in how and why they are established. They vary in composition, intended purpose, and means by which members exchange information and knowledge. Some CoPs were established as management initiatives, while others operated without being formally recognised as CoPs. In common, however, was the intention to facilitate learning and the exchange of information or knowledge; or to improve practice.

The question that follows is whether CoPs with such diversity in structure and function deliver the gains in performance for which they are being promoted. The findings from this review suggest that while early research on CoPs in the healthcare sector could not, and was not designed to answer this question, more recent research efforts have attempted to assess the impact of CoPs in improving quality of healthcare. Early indications from these efforts are that CoPs, on their own or as part of larger interventions, may have a role in improving healthcare performance.

With such indications emerging, it becomes even more important to know how best to, as suggested by Wenger and colleagues [7], cultivate CoPs to benefit their organisations. As indicated by Li and colleagues, bringing together a group of people and calling them a CoP does not mean that they will function as one [11]. There are factors that will influence and hinder the successes of these groups. A facilitating factor in one group may be a hindrance to another group working towards a different goal or under different circumstances. For example, included in this review was a research study where face-to-face meetings of members were found to be important to keep the energy levels of the group high [39]. In contrast, other papers reported on groups that met solely or largely using virtual methods and yet, functioned effectively [21,40]. Understanding how and why such differences determine success is essential in knowing how to facilitate and support such groups to maximise their potential.

This review has noted that CoPs can have a role in achieving a diverse range of outcomes including, but not limited to, gaining competencies following completion of basic training; breaking down professional, geographical and organisational barriers; sharing information; reducing professional isolation; and facilitating the implementation of new processes and technology (see Additional File 1). While this systematic review defined and examined outcomes in terms of a demonstrable change in work practice or outcomes, this does not imply that the other outcomes identified in the review are not important. Improving performance of healthcare organisations involves, and is achieved through, behaviour change among individuals working for the organisation, and many of the outcomes of the research studies reviewed in this paper reflect such change. Applying greater weight for these findings to be considered as evidence of the impact of CoPs improving healthcare practice requires that such claims and perceptions be assessed using more comprehensive methods such as triangulation, to confirm translation of such behavioural changes into practice.

A finding of this review is that there has been a noticeable increase in empirical research papers on CoPs being published in the healthcare literature since 2005, the end of the search period for the Li *et al *review. Importantly, researchers are increasing their efforts to examine the impact of CoPs in healthcare by attempting to quantify effectiveness. It is also noted that while in keeping with the original CoP concept, learning and knowledge exchange continues to be the focus, more recent literature suggests that CoPs in healthcare are being more targeted in their focus, specifically on sharing and promoting evidence-based best practice among practitioners. As expected, email and web-based communication systems are being utilised more often to facilitate communication.

None of the research identified in this review addressed sustainability of the benefits gained through facilitating or establishing CoPs in healthcare. Demonstrating sustainability requires longitudinal follow-up. When planning the implementation of CoPs, it is important that researchers and service providers recognise the need to establish baseline measures and indicators that will facilitate assessment of long-term effects.

There are limitations associated with this review. For example, grey literature was excluded from consideration. Much work on CoPs in business is contained in such reports not freely available in the public domain; and it is possible that a similar situation exists in healthcare. Identifying and including such non-academic literature may have provided an additional perspective to help our understanding of the role of CoPs in improving performance in healthcare. The exclusion of literature from other sectors may also be considered a limitation, however, given the importance of social and cultural context in determining the effectiveness of interventions such as CoPs, findings from other sectors may not be applicable to the healthcare sector.

Also not identified from this review is the fact that there may be other features of CoPs that determine their ability to influence change in healthcare. For example, it is recognised that typically, CoPs go through stages of development, starting at the potential stage where individuals, still loosely connected, begin discovering common ground. In the presence of favourable conditions, groups may progress from coalescence to more mature stages, ultimately forming a stewardship CoP [7]. Some groups do not progress beyond the early stages, while others may progress steadily through the stages. The time spent in each of these stages may also vary. These are all factors that need to be taken into consideration when assessing the effectiveness of CoPs. Included in this review were research studies that observed or studied the emergence of potential CoPs [21,23,45]. The outcome of interest at this stage would be the relationships that are forming rather than looking for a change in work practice.

## Conclusion

With such variations in form and function, cultivating CoPs to benefit healthcare organisations requires a flexible framework that will guide rather than prescribe their establishment and facilitation. Such a framework might take into consideration the findings of this review. While there is now a quantum of literature relating to CoPs in healthcare that can be drawn on to develop such a framework, the value of future research can be enhanced by taking into consideration the need to look beyond whether or not CoPs achieve a pre-defined outcome. There is a need to understand that CoPs are complex, multifaceted programs that operate using different models. Additionally, given that CoPs are used in healthcare to influence change in practice, which requires a change in practitioner behaviour, the social and cultural context within which they operate is likely to influence impact. If CoPs are to be cultivated to benefit healthcare organisations, future research needs to take into consideration this complex and varying nature of CoPs and adopt other methods more suitable for evaluating complex programs in healthcare. Realist evaluation is one such method that would address the gap in knowledge required to assess the role of CoPs in healthcare by exploring how, why and when CoPs facilitate improvements in healthcare performance [13].

## List of abbreviations

CoP: Community of practice; GP: General practitioner; NHS: National Health Service.

## Competing interests

The authors declare that they have no competing interests.

## Authors' contributions

GR, in consultation with all authors, led the process of developing the search strategy and undertaking the search. GR and JJP independently screened all identified abstracts using the exclusion and inclusion criteria, and shortlisted papers for detailed review. GR reviewed and extracted required information from all papers included in the review, except for those that employed ethnographic methods; these were reviewed by JJP. All authors contributed to the development of the search strategy and to the writing and review of the paper; and have read and approved the final manuscript.

## Pre-publication history

The pre-publication history for this paper can be accessed here:

http://www.biomedcentral.com/1472-6963/11/273/prepub

## Supplementary Material

Additional file 1 **Research methods used to study communities of practice in the healthcare sector, by research method, in chronological order**. A table summarising the research methods used in the studies included in the review. Click here for file
