# Supplementary material for: How and why are communities of practice established in the healthcare sector? A systematic review of the literature
Source: BMC Health Serv Res. 2011 Oct 14;11:273. doi: 10.1186/1472-6963-11-273 (PMC3219728; doi:10.1186/1472-6963-11-273)
Supplement: Additional file 1 — Research methods used to study communities of practice in the healthcare sector, by research method, in chronological order. A table summarising the research methods used in the studies included in the review. [file 1472-6963-11-273-S1.PDF]

Research methods used to study communities of practice in the healthcare sector, by research method, in chronological order

Reference and year of publication	Research method	Primary focus of research	Data collection method				Findings/conclusion from original paper
			Ethnography	Focus groups	Self-reported (interviews/ surveys)	Document review/other data sources	
**Qualitative research (n=24)**							
[27] 1999	Qualitative.	Observational exploration of processes by which novices become members of a CoP and senior members continue to learn.	Observations.		In-depth interviews.		Passing the qualifying exam was seen as the beginning of the process to gaining competency. Learning in the workplace was identified as being complex and influenced by many factors.
[35] 2000	Qualitative intervention study.	To engage research inexperienced nurse clinicians in research through participation in a workshop that was designed to apply the situated model of learning.			Feedback from participants.		Intervention = multifaceted workshop. Participants provided favourable feedback. Benefits of situated learning techniques were discussed and the authors concluded that while the situated learning model takes more time and effort for the learner and the instructor, it provides lasting benefits by fostering learning skills.

[20] 2002	Qualitative action research.	Facilitation of interagency collaboration.	Observations at meetings.		Interviews with participants.	Recordings of meetings.	The potential of the CoP to develop and improve local services, and to breakdown interagency and inter-professional boundaries and barriers, was identified. Factors that influence the development, functioning and maintenance of CoPs were discussed.
[33] 2002	Qualitative.	The potential for small groups of physicians to function as a CoP, which acts as a vehicle for sharing information, learning and practice change.		Focus groups with facilitators.	Interviews with facilitators and group members.		Groups provided friendly atmosphere to build professional relationships. Relationships were supportive and valued. Members reported that they listened, reflected, shared experiences and received feedback. While physicians recalled changes to diagnostics, therapeutics, screening and patient education, physicians did not feel that the sessions dramatically changed their practice.

[24] 2002	Qualitative case study.	Ability to influence radical innovation (for treatment of prostate cancer).			Interviews with managers pivotal in mobilising the innovation, and sales representatives.		CoPs helped mobilise the innovation by; enabling the innovation to transcend professional boundaries, aligning commercial interest with the values of the professional group, and providing incentives for the sales staff to get involved in the innovation project.
[16] 2003	Qualitative case study.	Processing and application of knowledge.	Observation and tape recording of CoP meetings.		Interviews.	Review of CoP notes and outputs.	The relationships between the type of knowledge used, the knowledge behaviours of the members, and the roles and agendas of the individual members, were complex. Interpretation and uptake of knowledge was strongly influenced by personal professional and political agenda.
[17] 2004	Qualitative.	A theoretical model constructed on the way in which evidence and information is built into clinical and policy decisions.	Non-participant observation.		Semi-structured formal and informal interviews.	Document review.	Practitioners acquired knowledge from sources they trusted – their CoPs, and rarely from formal sources of information.

[21] 2004	Qualitative case study.	The knowledge exchange process in an informal email network CHAIN (Contact, Help, Advice and Information Network).	Observed the network in operation.	Three virtual asynchron ous focus group discussions using private email groups.	Interviews with two core staff of the CHAIN network and members whose messages had been tracked.	Tracking of email messages, qualitative analysis of message postings from focus groups, feedback to the service.	CHAIN was seen as an example of how informal social networks help personalise and make knowledge meaningful within an organisation but also cross organisational and professional boundaries.
[39] 2004	Qualitative case study.	The potential for CoPs to facilitate the development of integration information systems in health.			Individual and group interviews and web-based survey.		The development of the CoP was seen as demonstration of the power and effectiveness of CoPs in accelerating the development of complex information systems. The CoP was highly dependent on face-to-face meetings to keep the energy alive.
[18] 2005	Qualitative.	Observed distribution of work and knowledge across medical-nurse boundary.	Observation +/- debrief with anaesthetist immediately post session.		Interviews with 20 staff selected to reflect the range of roles, skills and expertise,		Demonstrated constraints relating to professional boundaries within multi-professional CoPs. Legitimate access and participation was stratified by professional identity.
[19] 2005	Qualitative action research.	To develop a practitioner-led description of gerontological nursing			Group interviews	Review of online discussions.	The CoP helped bridge the divide between practitioners and academics to define gerontological nursing in a way that it reflected the real world of practice.

[46] 2005	Qualitative descriptive study	The experience of using the virtual college; and the extent to which the description of gerontological nursing and the first best practice statement on nutrition had influenced practice.		Semi-structured telephone interviews.	Access to the virtual college varied and was limited by access to computers. All 15 participants (half of the nurses participating in the project) self-reported that the Nursing Demonstration Project (NDP) that included a CoP had influenced their own practice. The CoP was part of the NDP and included nurses and academics collaborating to identify practice development priorities.

[22] 2006	Qualitative descriptive.	Design an online system for sharing and discussing critical incidents.	Observations of audit meetings and anaesthetic practice.	Semi-structured interviews.	Analysis of incident reporting forms or copies of outputs from the system.	User involvement in the design of the system was seen as favourable. Although only seven incidents were reported over the six-month pilot period (of an estimated 1434 incidents expected), participants perceived the system to be successful in terms of the quality and usefulness of the discussions. A possible explanation provided by the authors for the lower than expected number reported was that only incidents judged to have broader educational relevance were posted.

[40] 2007	Qualitative case study.	Implement evidence-based care in emergency departments.	Describes the history leading up to the establishment of the emergency department (ED) CoP. No data collection undertaken. Audit of indicators was referred to in relation to one of the implementation initiatives aimed at improving evidence-based pain management in emergency care.	The paper included a summary of the first major implementation activity initiated through the ED CoP. This mental health emergency care interface project achieved statistically significant improvements in target indicators including a reduction in 'did not wait' rate for mental health presentations, and an increase in the number of patients discharged within 4 hours of initial presentation. Also reported was that 1995 resources were downloaded from the project website with clinical assessment tools, protocols and process maps being the most popular downloads.

[31] 2007	Qualitative case study.	Increase in research capacity for tobacco control.	Describes the model. Data collection methods were not specified.		The following achievements were listed: Increase in attendance at annual symposiums by 220%; Rapid infusion of students and scientists into field of tobacco control; Renewed commitment by experienced researchers to collaborate; New multidisciplinary teams of people being established and receiving seed grants to work on problems of mutual priority; Increase in communication exchange; Policy makers and researchers volunteering to be part of the policy SWAT initiative; increased number of data sets being deposited in the data repository.
[34] 2007	Qualitative.	Encourage interdisciplinary end-user input into the endorsement of clinical guidelines on low-back pain management.		Comments received on the website were systematically analysed. Online survey conducted three months after release of guidelines to obtain their evaluation.	The majority of respondents to the survey thought that they had sufficient opportunity to contribute; agreed with the contents of the guidelines; and had distributed it to colleagues. The authors identified the limitation of this study in addressing only one factor that influences adherence to guideline recommendations.

[44] 2007	Qualitative case study.	Implementing information technology in healthcare.	Observation of the Electronic Management Plan (EMP) steering committee and clinicians involved in the development and pilot test of EMP.	Semi-structured interviews with clinicians.	The clinician planning group (likened to a CoP) was formed by the steering committee. The authors state that this group was effective in accumulating knowledge about ongoing developments, and the experiences and difficulties with the system and functionality. Following a restructuring and loss of official recognition, this group lost enthusiasm and the knowledge acquired by the group was underutilised.
[43] 2007	Qualitative action research.	Explanation and description of occupational therapy practice.		In-depth interviews.	The difficulty in explaining and defining occupational therapy practice was identified. The feedback provided to the occupational therapists as part of the action research design, encouraged the group to undertake in-depth, reflective action within a CoP model and to make changes to their practice. The authors propose to report on the effectiveness of these actions in a later publication.

[42] 2008	Qualitative case study.	Decrease professional isolation for individuals working in small, less resourced clinical units.	Survey of Listserv members.	Listserv activity.	Listserv membership had increased since establishment. Activities focussed on requests for, and exchange of information such as guidelines and policies. The majority of the 26% respondents self reported the postings to be relevant, and over 25% of respondents forwarded the newsletter to a colleague. Medical educators were the most active participants and were more likely to respond to questions, while rural members were more likely to pose a question compared with responding.
[36] 2008	Qualitative descriptive case-study.	Improved clinical practice.	Unclear whether survey or interview. Authors stated that at the beginning of each session, facilitators ascertained which learnings from the previous session had been implemented.		Of the 200 identified learnings, 55% were self-reported as being implemented. The authors identify the limitation in not verifying through audits or follow-up that the learnings were actually implemented. Cognitive and behavioural barriers were the most frequently identified category of barriers.

[30]<br>2008	Qualitative descriptive.	Development of an implementation model to improve delivery of cancer surgery services. The model needed to be flexible, adaptable, manageable, and open to evaluation.		Interviews with clinicians and key stakeholders and surveys of clinicians. Note that these are data collection methods proposed in presenting the framework. It is not clear from the paper what data collection methods were used in testing of the CoP model or whether the model was tested.	The authors state in the summary that the preliminary results demonstrated that physicians participating in the CoP had high levels of satisfaction with clinical leadership and knowledge transfer.

[23] 2008	Qualitative descriptive.	Impact of clinical leadership program on perceived clinical leadership capabilities, and clinical practice.	Observation.	Focus groups with those that had completed the program in the previous 18 months.	Self-administered questionnaire and in-depth interviews.		Effects on clinical practice reported include ability to use different strategies at different times for different situations; more proactive in dealing with work challenges; significant impact on team effectiveness and thus work outcomes' willingness to embrace change; and emergence of CoPs. Leadership skills that were developed were also listed in the paper.

[15] 2009	Qualitative descriptive.	Sustainable service improvement (fill communication gaps and reduce duplication)		Survey of members.		Response rate to the survey was low (27%). Respondents perceived the CoPs to have provided members with the opportunity to network and collaborate, develop new knowledge, transfer good practice, discuss ideas, provide peer-support for practical issues, and to speed up learning. Authors conclude that CoPs are effective in breaking down cultural barriers to sharing between professions and agencies, but that the benefits are indirect and can only be correlated with service improvement (as opposed to being directly attributed).
[29] 2009	Qualitative – descriptive.	The merits of an online media in the exchange of knowledge between urban and rural clinicians.		Survey of all participants.	Content analysis of online discussion boards.	Participants self-reported that the virtual CoP provided access to new sources of knowledge and resources.

Quantitative research (n=7)

[45]<br>2006	Quantitative case study.	Potential emergence of a CoP following the bringing together of geographically dispersed individuals known to work in the area of web-assisted tobacco intervention.		Knowledge, attitude, expectations and learnings were measured using a short-instrument administered three week's post initial meeting.	Relationships important for knowledge and information exchange were examined using social network analysis methods.	Based on emails discussions that followed the initial meeting, the authors claimed evidence of the group being in coalescence phase of a CoP and poised to move into the mature phase.
[37]<br>2006	Quantitative. A component of a RCT. Randomisation was not to allocate to a study group but to determine where treatment would be initiated.	i) Process adherence<br>ii) Catheter-related blood stream infection rate.			Reporting of recorded outcome measures.	The direct role of the hospital leadership and the local CoP was recognised in achieving a 50% reduction in catheter-related blood stream infections. These factors were believed to have facilitated the cooperation of physicians.

[32] 2007	Quantitative intervention trial.	Shift in culture from blame to patient safety. Number of liability claims. Change in core clinical knowledge.		Test of clinical knowledge and culture change.	Data on number of insurance claims as provided by The Healthcare Insurance Reciprocal of Canada.	Intervention = obstetrics patient safety program. Clinical core knowledge increased significantly and the difference between disciplines (nurses, family physicians, midwives and obstetricians) narrowed. Culture change assessment suggested a change in culture with patient safety becoming a greater priority for all. Scores for learning, open communication, and team working improved, and based on liability claims data, liability costs reduced.

[26] 2008	Quantitative intervention study.	Promotion of evidence-based practice.	CoP members invited to participate.	Facilities and patient audits.	Nursing work-index questionnaires completed by CoP members.	Intervention = implementation of a model designed to promote evidence-based practice. The virtual college was seen to be a supportive resource as it provided easy and rapid access to knowledge and other experts. The facilities audit identified improvements in developing local guidelines and policies and the use of validated tools. The patient audit suggested more relationship-oriented patient care, improvements in assessment of nutritional needs and nutrition audits for older persons; and increased use of screening tools and comprehensive assessments. Greater involvement of the older person was also identified. The nursing work-index suggested nurses experiencing greater autonomy in the workplace.

[38] 2008	Quantitative intervention trial.	Improve colorectal, breast, and cervical cancer screening rates.		Cancer screening rates reported by the participating community health centres.	Intervention = learning model to improve cancer screening rates, and included a range of activities. Activities undertaken by the CoPs varied across the four study sites, and one CoP did not develop local links. Self-reported screening rates improved, but still remained lower than self-reported national rates.
[41] 2009	Quantitative intervention trial	Improving quality of referral letters written by general practitioners (GPs) to specialist medical practitioners.		Referral letters were submitted for scoring by GPs. The scoring was done by a researcher with medical training, using benchmarks set by the members of the CoP.	Intervention = Establishing a CoP of GPs in rural and metropolitan locations. Attrition rate was high with ten of the 15 GPs not completing the audit cycle. The authors reported a significant improvement in the scores following the establishment of the CoP. The challenges associated with implementing this research were discussed.

[28] 2009	Quantitative Randomised trial	Uptake of an evidence-based outcome measurement tool to monitor client response to treatment and to measure service outcomes.		Questionnaires to assess knowledge change, use of supports, satisfaction with supports, and practice change.	Child and Adolescent Functional Assessment Scale (CAFAS) database.	Practitioners were randomised to a group supported by a CoP or ‘practice as usual’ group. While the CoP-supported group did not differ significantly from the comparison group in terms of self-reported practice change, this group demonstrated greater use of the tool in the real world practice context and better knowledge of the tool at the end of one year.
**Systematic reviews (n=2)**						
[11] 2009	Systematic review	Evaluation of effectiveness of CoPs in improving uptake of best practice and mentoring new practitioners.			Systematic review of literature.	The authors did not find any paper in the health sector that met their eligibility criteria, and concluded that the effectiveness of CoPs in healthcare remains unclear.

[14] 2009	Systematic review	Identifying regional collaborations in surgical practice examining practices related to quality improvement.		Systematic review of literature.	Seven papers on collaborative initiatives were reviewed. Authors reported that all of these papers reported improvements in quality improvement measures. They concluded that a CoP framework can be used as a model for collaboration to improve quality of care and to foster professional development.
